# Glycosuria in War Traumatisms

**Published:** 1918-07

**Authors:** 


**Glycosuria in War Traumatisms.** F. Rathery, Le Prog. Med., Oct. 27, 1917, found 60 cases in 1,412 wounded (4.17%). It was more frequent in cases of multiple wounds, amounted to 2-20, rarely 30 grams of sugar a day, usually lasted only 2-3 days but in one case 15 days and in another several months. It never amounted to a true diabetes.

